# Routine Nutritional, Inflammatory, and Redox-Related Biomarker Abnormality Burden Is Associated with Lower 12-Week Physical Performance After Cholecystectomy in Rehabilitation Outpatients

**DOI:** 10.3390/antiox15101306

**Published:** 2026-10-09

**Authors:** Geunhyeok Yang, Eo Jin Park

**Affiliations:** 1 Division of Hepatobiliary Surgery and Transplantation, Department of Surgery, Kyung Hee University Hospital at Gangdong, Kyung Hee University College of Medicine, Seoul 05278, Republic of Korea; 2 Department of Rehabilitation Medicine, Kyung Hee University Hospital at Gangdong, Kyung Hee University College of Medicine, Seoul 05278, Republic of Korea

**Keywords:** cholecystectomy, routine biomarker abnormality burden, rehabilitation, Short Physical Performance Battery, grip strength, 6-min walk test, physical performance, retrospective cohort

## Abstract

Simple tools for characterizing functional vulnerability after cholecystectomy are lacking, particularly among patients referred for outpatient rehabilitation. We examined whether a cumulative count of routine nutritional, inflammatory, and redox-related biomarker abnormalities at the first physical medicine and rehabilitation (PM&R) visit was associated with 12-week physical performance. This single-center retrospective cohort included 184 adults assessed 14–180 days after cholecystectomy. The exposure counted abnormalities in zinc, 25-hydroxyvitamin D, homocysteine, albumin, and neutrophil-to-lymphocyte ratio. The primary outcome was the 12-week Short Physical Performance Battery (SPPB) total score. After multivariable adjustment, each additional abnormality was associated with a 0.49-point lower 12-week SPPB score (β = −0.49, 95% confidence interval = −0.80 to −0.18; *p* = 0.002). Supportive analyses showed higher odds of low physical performance, lower odds of substantial SPPB improvement, lower grip strength, and a shorter 6-min walk distance. The additional explanatory value was modest (incremental R^2^ = 0.03). A higher routine biomarker abnormality burden may characterize postoperative vulnerability relevant to rehabilitation assessment, but the count is not a validated prediction or treatment selection tool and requires prospective validation.

## 1. Introduction

Cholecystectomy is commonly performed for benign gallbladder disease and is usually followed by uncomplicated recovery [1,2,3,4]. Nevertheless, postoperative recovery is not uniform: a clinically relevant subset experiences reduced physical performance, persistent pain, or delayed return to usual activity [2,3]. For patients who reach outpatient rehabilitation, the central clinical problem is not operative success itself but identifying functional vulnerability that may delay recovery [5]. Because this selected population may reflect both incomplete surgical recovery and preexisting vulnerability, a simple measure available at the first rehabilitation visit could help characterize patients who may need closer functional assessment and follow-up [5,6]. This makes the rehabilitation entry visit a clinically relevant assessment point even though cholecystectomy is generally considered a lower-risk operation [5,6].

Physical performance after surgery reflects overlapping domains of baseline mobility, pain, comorbidity, nutritional reserve, inflammation, and redox-related physiologic stress [5,6]. The five routinely available biomarkers evaluated here were therefore considered indicators of three broad domains: nutritional reserve, inflammatory activity, and redox-related physiology [5,6,7], with component-specific rationale supported by prior literature [8,9,10,11]. No component is specific to a single domain, and none is a direct measure of oxidative stress. A cumulative count may nevertheless summarize multidomain physiologic vulnerability more pragmatically than any isolated result [6,7]. Because these measurements were obtained in routine clinical care, the count may provide a practical bridge between laboratory findings and rehabilitation assessment [5,7].

Existing cholecystectomy studies have focused on postoperative complications, risk prediction, and preoperative physical activity in relation to recovery [12,13,14]. These studies inform perioperative risk and recovery but do not evaluate whether a cumulative burden of routine nutritional, inflammatory, and redox-related abnormalities at entry to outpatient rehabilitation is associated with subsequent standardized physical performance [6,7,14]. This gap is clinically relevant because the first physical medicine and rehabilitation (PM&R) outpatient visit occurs after the immediate postoperative period, when functional limitation can be assessed directly and routinely available biomarkers can be interpreted alongside baseline performance [5,14]. Unlike specialized oxidative stress assays, a pragmatic count based on routine laboratory tests is feasible in retrospective clinical practice while remaining a multidomain vulnerability indicator rather than a direct oxidative stress measure [7,15,16].

Therefore, we aimed to determine whether the routine biomarker abnormality count at the first PM&R outpatient rehabilitation visit after cholecystectomy was associated with 12-week physical performance. The 12-week Short Physical Performance Battery (SPPB) total score was selected as the primary outcome because it is a standardized measure of lower-extremity physical performance and captures short-term functional status after a consistent rehabilitation interval [17,18]. We also evaluated supportive outcomes, including low 12-week physical performance, substantial SPPB improvement, grip strength, 6-min walk distance, and pain. We hypothesized that a higher count would be associated with less favorable 12-week outcomes after adjustment for baseline physical function, pain, timing, demographic characteristics, and comorbidity. The count was evaluated as a simple vulnerability indicator rather than as a validated prediction or treatment selection tool.

## 2. Materials and Methods

### 2.1. Study Design and Participants

This single-center retrospective cohort study screened adults who attended the physical medicine and rehabilitation (PM&R) outpatient rehabilitation clinic after cholecystectomy between 1 January 2017, and 31 December 2024. The source population was restricted to adults who attended the PM&R outpatient rehabilitation clinic after cholecystectomy; therefore, the cohort does not represent all patients undergoing cholecystectomy. The analytic dataset did not contain a standardized referral indication or referral-criteria variable; so, uniform referral criteria could not be reconstructed retrospectively. Eligibility required age ≥ 19 years, a benign cholecystectomy indication, a first outpatient rehabilitation episode after the index cholecystectomy, a baseline PM&R visit 14–180 days after surgery, complete baseline biomarker data, and a 12-week reassessment within 84 ± 14 days of the baseline visit. The 14-day lower bound was selected to exclude the immediate perioperative phase, when acute surgical recovery could dominate functional assessment, whereas the 180-day upper bound was selected to restrict the cohort to a postoperative rehabilitation episode rather than later chronic functional care. The surgical institution was not used as a selection criterion because this study focused on functional status at entry to rehabilitation and included patients whose operations may have been performed at different institutions.

Medical records of 382 adults were screened. Exclusion criteria were inadequate documentation related to cholecystectomy; a first PM&R visit outside 14–180 days after surgery; no 12-week reassessment within the prespecified window; incomplete baseline biomarker data; a non-benign indication or concomitant major abdominal surgery; a preexisting neurological or musculoskeletal condition affecting performance assessment; a repeated rehabilitation episode after the same cholecystectomy; age < 19 years; or pregnancy. Inadequate documentation referred to records in which the available information was insufficient to confirm eligibility for the index cholecystectomy episode or its temporal relationship to the rehabilitation episode. Concomitant major abdominal surgery referred to an additional abdominal operation considered likely to have a substantial independent effect on postoperative recovery. Preexisting neurological or musculoskeletal conditions were considered exclusionary when the clinical record indicated an independent condition likely to compromise interpretation of the study performance assessments. The screening log retained exclusion categories but not the individual missing elements, diagnoses, or procedure details for excluded patients; therefore, more granular lists could not be reconstructed reliably. Concomitant major abdominal surgery was excluded because it could independently dominate postoperative recovery, whereas neurological or musculoskeletal conditions were excluded because they could invalidate interpretation of the functional assessments. After exclusions, 184 patients comprised the final analytic cohort (Figure 1).

The study protocol was reviewed and approved by the Institutional Review Board of Kyung Hee University Hospital at Gangdong (IRB No. 2026-08-010; approval date: 31 August 2026). The requirement for informed consent was waived because this study was a retrospective review of de-identified clinical data.

### 2.2. Data Collection and Variables

All laboratory biomarkers used to define the exposure were measured at the institutional laboratory of Kyung Hee University Hospital at Gangdong and were abstracted from clinically obtained results within ±14 days of the baseline PM&R visit. The analytic dataset retained one abstracted value per biomarker within this window. Assay-platform identifiers, institutional reference intervals, laboratory-result timestamps needed to reconstruct the selection among multiple same-window measurements, and preoperative values were not retained and therefore could not be reported reliably. The neutrophil-to-lymphocyte ratio was calculated by dividing the absolute neutrophil count by the absolute lymphocyte count from the same complete blood count.

Outpatient rehabilitation was delivered as usual care and individualized according to the treating physician’s assessment. Session number, frequency, treatment components, and intensity were not recorded consistently enough for reliable analysis and were therefore not included in the primary model. Because rehabilitation exposure may directly influence 12-week SPPB, grip strength, and walking distance, its absence was considered a potential source of residual confounding rather than evidence that treatment exposure was unimportant.

Clinical and laboratory data were obtained from electronic medical records. Baseline variables included age, sex, body mass index, calendar year of the baseline PM&R visit, surgery-to-PM&R visit interval, acute cholecystitis indication, diabetes mellitus, Charlson Comorbidity Index, baseline SPPB total score, baseline grip strength, baseline 6-min walk test distance, and baseline pain Numeric Rating Scale (NRS) score [17,19,20,21]. The surgery-to-PM&R visit interval was calculated from the date of cholecystectomy to the first outpatient rehabilitation visit. The calendar year of the baseline PM&R visit was included to account for possible secular changes in referral patterns and outpatient rehabilitation practice during the 8-year study period.

The routine biomarker abnormality count comprised routinely available measures spanning nutritional, inflammatory, and redox-related domains [10,11,22], with component-specific thresholds supported by prior biomarker literature [22,23,24]; the five components and their thresholds are defined once in Section 2.4. Surgical approach, postoperative length of stay, postoperative complications, readmission, perioperative management, dietary intake, preoperative physical activity, and rehabilitation exposure were not available as sufficiently complete analytic variables. This study therefore focused on vulnerability at the first rehabilitation visit rather than on the perioperative course while recognizing that unmeasured surgical severity could confound the observed association.

### 2.3. Outcome Measures

The SPPB was administered using the standard balance, gait-speed, and repeated-chair-stand protocol [17,18,25]. Grip strength was measured three times per hand with a digital hand dynamometer (Grip-D, T.K.K. 5401; SANKA Co., Ltd., Niigata, Japan), and the highest value was analyzed [20,26]. The 6-min walk test followed a standardized clinical protocol [21], and pain was rated at the baseline and 12-week PM&R assessments on an 11-point NRS from 0 (no pain) to 10 (worst imaginable pain) [27,28].

Pain assessment was performed as part of the routine clinical evaluation. The analytic dataset did not contain the timing of analgesic administration or immediate preassessment analgesic use; so, these factors could not be evaluated retrospectively. Hand dominance was also unavailable. Functional assessments were performed using the standardized protocols described above. The analytic dataset did not contain assessor identity or documentation of study-specific evaluator training, calibration, blinding, or inter-rater reliability testing; therefore, these features could not be evaluated retrospectively.

The primary outcome was the 12-week SPPB total score. The 12-week interval was selected to capture short-term functional status after a consistent period of outpatient follow-up. SPPB scores range from 0 to 12, with higher scores indicating better physical performance [17,18]. Low 12-week physical performance was defined as SPPB ≤ 9 [18,29]. Substantial improvement was defined as an increase of ≥2 points from baseline [30,31]; this was a pragmatic supportive threshold rather than a definitive minimal clinically important difference [30,31].

Supportive continuous outcomes included 12-week grip strength, 12-week 6-min walk test distance, and 12-week pain NRS score. Grip strength was summarized in kilograms, the 6-min walk test distance was summarized in meters, and pain NRS scores were summarized using the 11-point scale [20,21,27]. These measures were routinely assessed in patients receiving outpatient rehabilitation in this cohort and were analyzed as supportive outcomes according to the primary/supportive outcome hierarchy used in this study.

### 2.4. Exposure/Predictor Measures

The primary exposure was an unweighted count of five routine nutritional, inflammatory, and redox-related biomarker abnormalities: zinc < 70 μg/dL, 25-hydroxyvitamin D < 20 ng/mL, homocysteine ≥ 15 μmol/L, albumin < 4.0 g/dL, and a neutrophil-to-lymphocyte ratio ≥ 3.0 [22,24]. The zinc, vitamin D, and homocysteine cutoffs were selected as established clinical abnormality thresholds. Albumin < 4.0 g/dL was used as a pragmatic marker of low albumin status, not conventional hypoalbuminemia [23,32], and a neutrophil-to-lymphocyte ratio ≥ 3.0 was used as a practical threshold for elevated inflammatory burden rather than as a universal diagnostic cutoff. The count ranged from 0 to 5 and was intended as a simple multidomain burden indicator, not a validated score or a direct measure of oxidative stress [6,7,15].

For descriptive analyses, patients were grouped as 0–1, 2, or 3–5 abnormalities. These categories reflected the observed distribution (*n* = 23, 49, 57, 38, 15, and 2 for counts 0–5, respectively) and avoided unstable estimates at sparse extremes. A high routine biomarker abnormality burden was defined descriptively as ≥3 abnormalities. The continuous count was used in the primary model and represented the average adjusted difference per additional abnormality. This specification assumes an approximately constant average increment and equal unit weighting across components; categorical estimates and adjusted count-specific predictions were therefore examined to assess the shape of the association and were not interpreted as proving a dose–response relationship. The primary covariate set included baseline SPPB total score, baseline pain NRS score, age, sex, body mass index, calendar year of the baseline PM&R visit, surgery-to-PM&R visit interval, acute cholecystitis indication, diabetes mellitus, and the Charlson Comorbidity Index [17,19].

### 2.5. Statistical Analysis

Continuous variables were summarized as means with standard deviations or medians with interquartile ranges, and categorical variables were summarized as frequencies and percentages. Baseline characteristics across abnormality groups were compared using one-way analysis of variance, the Kruskal–Wallis test, or the chi-square test, as appropriate. Unadjusted 12-week rehabilitation outcomes across abnormality groups were summarized descriptively and compared using one-way analysis of variance or the chi-square test, as appropriate.

The primary association between the routine biomarker abnormality count and the 12-week SPPB total score was examined using multivariable linear regression with HC3 robust standard errors. Regression coefficients were interpreted as adjusted mean differences in the 12-week SPPB total score per additional abnormality. Model performance was evaluated using R^2^, adjusted R^2^, root mean square error (RMSE), mean absolute error (MAE), and variance inflation factors (VIFs). The incremental R^2^ attributable to the count beyond the clinical covariate model was also calculated. Internal validation used 5000 bootstrap resamples, with optimism-corrected R^2^, optimism-corrected RMSE, and the calibration slope reported. To display model shape, adjusted marginal mean 12-week SPPB scores were calculated for counts 0–5 by setting the count to each level for all participants and averaging the model-based predictions; 95% confidence intervals were based on the HC3 covariance matrix, and the observed number of participants at each count was displayed.

Supportive logistic regression models were used to evaluate low 12-week physical performance and substantial 12-week SPPB improvement. The binary supportive models used the primary covariate set. Model discrimination and overall prediction error were evaluated using the area under the receiver operating characteristic curve (AUROC) and the Brier score, respectively. Supportive linear regression models with HC3 robust standard errors were used to evaluate 12-week grip strength, 12-week 6-min walk test distance, and 12-week pain NRS score. The grip strength and 6-min walk test models included the corresponding baseline performance measure, baseline pain NRS score, and the same clinical covariates. The pain model included the baseline pain NRS score, baseline SPPB total score, and the same clinical covariates.

Robustness analyses included categorical modeling of the abnormality count, trend modeling across abnormality count categories, median quantile regression, ordinal logistic regression using tertiles of the 12-week SPPB total score, models using conventional and HC3 robust standard errors, bootstrap internal validation, influence diagnostics after excluding observations with Cook’s distance > 4/N, centered interaction analyses between abnormality count and the surgery-to-PM&R visit interval, a model excluding calendar year, spline-adjusted models for age and the surgery-to-PM&R visit interval, an analysis excluding patients with acute cholecystitis, component-wise continuous biomarker analyses, leave-one-component-out analyses, and VIF assessment. All analyses were performed using R software (version 4.2.3; R Foundation for Statistical Computing, Vienna, Austria).

All analyses were conducted as complete-case analyses because patients with incomplete baseline biomarker data were excluded during cohort assembly. No imputation was performed. The screening log retained exclusion counts but not a complete set of covariates for excluded patients; therefore, a reliable included-versus-excluded comparison could not be performed. This limitation was considered when interpreting selection bias. Supportive analyses were considered exploratory; therefore, no formal adjustment for multiple comparisons was applied, and supportive *p* values were interpreted descriptively.

## 3. Results

The participant selection process is shown in Figure 1. Of the 382 screened adults, 198 were excluded, most commonly because the first outpatient rehabilitation visit occurred outside 14–180 days after surgery (*n* = 55), a 12-week reassessment was unavailable within the prespecified window (*n* = 39), or baseline biomarker data were incomplete (*n* = 38). The final analytic cohort included 184 patients who represented their first outpatient rehabilitation episode after cholecystectomy and completed the prespecified 12-week reassessment. Because complete covariate data for excluded patients were not retained, baseline comparisons between included and excluded groups could not be performed reliably.

Baseline characteristics according to the number of routine biomarker abnormalities are summarized in Table 1. The mean age was 63.2 ± 10.3 years, and 83 patients (45.1%) were male. The median surgery-to-PM&R visit interval was 66 days (interquartile range [IQR], 44–86 days). Acute cholecystitis was the indication for surgery in 119 patients (64.7%), diabetes mellitus was present in 43 patients (23.4%), and the median Charlson Comorbidity Index was 2 (IQR, 1–3). Across abnormality groups, age (*p* = 0.034), surgery-to-PM&R visit interval (*p* = 0.002), acute cholecystitis indication (*p* < 0.001), and diabetes mellitus (*p* < 0.001) differed significantly. The baseline SPPB total score was lower in the 3–5 abnormality group than in the 0–1 and 2 abnormality groups (6.2 ± 1.6, 8.2 ± 1.7, and 7.5 ± 1.7, respectively; *p* < 0.001). Baseline grip strength (*p* = 0.048), baseline 6-min walk test distance (*p* < 0.001), and baseline pain NRS score (*p* < 0.001) also differed across groups.

Table S1 presents the distribution of the five biomarker components. Zinc deficiency was present in 68 patients (37.0%), vitamin D deficiency in 94 patients (51.1%), hyperhomocysteinemia in 48 patients (26.1%), low-albumin status in 60 patients (32.6%), and a high neutrophil-to-lymphocyte ratio in 77 patients (41.8%). The number of routine biomarker abnormalities had a mean of 1.9 ± 1.2 and a median of 2 (IQR, 1–3). The distribution was 0 abnormalities in 23 patients (12.5%), 1 abnormality in 49 patients (26.6%), 2 abnormalities in 57 patients (31.0%), 3 abnormalities in 38 patients (20.7%), 4 abnormalities in 15 patients (8.2%), and 5 abnormalities in 2 patients (1.1%). A high routine biomarker abnormality burden, defined as ≥3 abnormalities, was present in 55 patients (29.9%).

Unadjusted 12-week rehabilitation outcomes are shown in Table S2. The overall 12-week SPPB total score was 8.9 ± 2.3, with values of 10.2 ± 1.6 in the 0–1 abnormality group, 8.5 ± 2.3 in the 2 abnormality group, and 7.5 ± 2.3 in the 3–5 abnormality group (*p* < 0.001). The mean change in SPPB total score from baseline to 12 weeks was 1.5 ± 1.9 points overall (*p* = 0.016 across groups). Low 12-week physical performance occurred in 101 patients (54.9%) overall and increased across abnormality groups from 23 patients (31.9%) in the 0–1 abnormality group to 34 patients (59.6%) in the 2 abnormality group and 44 patients (80.0%) in the 3–5 abnormality group (*p* < 0.001). Substantial SPPB improvement occurred in 86 patients (46.7%) and did not differ significantly across groups in the unadjusted comparison (*p* = 0.411). The 3–5 abnormality group also had lower 12-week grip strength, shorter 12-week 6-min walk test distance, and higher 12-week pain NRS score than the lower abnormality groups.

Table 2 shows the primary multivariable linear regression model for the 12-week SPPB total score. After adjustment for baseline SPPB total score, baseline pain NRS score, age, sex, body mass index, calendar year of the baseline PM&R visit, surgery-to-PM&R visit interval, acute cholecystitis indication, diabetes mellitus, and the Charlson Comorbidity Index, each additional routine biomarker abnormality was associated with a lower 12-week SPPB total score (β = −0.49, 95% CI = −0.80 to −0.18; *p* = 0.002). Baseline SPPB total score was also associated with a higher 12-week SPPB total score (β = 0.57, 95% CI = 0.41 to 0.73; *p* < 0.001). Baseline pain NRS score, age, body mass index, calendar year, surgery-to-PM&R visit interval, acute cholecystitis indication, diabetes mellitus, and the Charlson Comorbidity Index were not statistically significant in the primary model. Male sex showed a positive regression coefficient with borderline statistical significance (β = 0.51, 95% CI = −0.03 to 1.04; *p* = 0.064).

The primary model had an R^2^ of 0.46, an adjusted R^2^ of 0.42, an RMSE of 1.70, an MAE of 1.39, and an overall model *p* value of <0.001. The maximum VIF was 2.08. The incremental R^2^ after adding the abnormality count to the clinical covariate model was 0.03 (*p* = 0.002). Bootstrap internal validation yielded an optimism-corrected R^2^ of 0.39, an optimism-corrected RMSE of 1.82, and a calibration slope of 0.94.

Supportive outcome models are summarized in Table 3 and Figure 2. Because these analyses were supportive and were not adjusted for multiple comparisons, they were interpreted as providing secondary context for the primary SPPB model. Each additional routine biomarker abnormality was associated with higher odds of low 12-week physical performance (OR = 1.87, 95% CI = 1.24–2.83; *p* = 0.003; AUROC = 0.80; Brier score = 0.177) and lower odds of substantial SPPB improvement (OR = 0.67, 95% CI = 0.45–0.99; *p* = 0.042; AUROC = 0.73; Brier score = 0.207). In supportive linear regression models, each additional abnormality was associated with lower 12-week grip strength (β = −1.02 kg, 95% CI = −1.67 to −0.37; *p* = 0.002; adjusted R^2^ = 0.79; RMSE = 3.53 kg) and a shorter 12-week 6-min walk test distance (β = −19.20 m, 95% CI = −27.43 to −10.98; *p* < 0.001; adjusted R^2^ = 0.75; RMSE = 44.48 m). The association with the 12-week pain NRS score was positive but did not reach conventional statistical significance (β = 0.26, 95% CI = −0.01 to 0.52; *p* = 0.054; adjusted R^2^ = 0.37; RMSE = 1.28).

Full coefficients for the supportive logistic and linear regression models are provided in Tables S3 and S4. Robustness analyses for the primary association are summarized in Table S5. In the categorical abnormality model, the adjusted difference in the 12-week SPPB total score was β = −1.09 (95% CI = −1.78 to −0.41; *p* = 0.002) for 2 abnormalities versus 0–1 abnormalities and β = −1.08 (95% CI = −1.94 to −0.21; *p* = 0.015) for 3–5 abnormalities versus 0–1 abnormalities. Because these categorical estimates were similar, the categorical analysis was not interpreted as demonstrating a clear adjusted dose–response gradient. The ordinal logistic regression model using tertiles of the 12-week SPPB total score showed lower odds of being in a higher tertile with each additional abnormality (OR = 0.63, 95% CI = 0.43–0.91; *p* = 0.013). Median quantile regression yielded an attenuated estimate (β = −0.19, 95% CI = −0.58 to 0.20; *p* = 0.339). Component-wise continuous biomarker analyses did not show statistically significant associations for most individual biomarkers (Table S6). Leave-one-component-out analyses generally retained negative associations for the 4-item abnormality count, with estimates ranging from β = −0.36 to β = −0.60 (Table S7). VIFs in the primary model ranged from 1.03 to 2.08 (Table S8).

Figure 3 presents adjusted marginal mean 12-week SPPB scores for each routine biomarker abnormality count. The estimated scores were 9.80 (95% CI = 9.15–10.45), 9.30 (95% CI = 8.91–9.69), 8.81 (95% CI = 8.55–9.08), 8.32 (95% CI = 7.90–8.74), 7.83 (95% CI = 7.15–8.52), and 7.34 (95% CI = 6.36–8.32) for counts 0–5, respectively; the corresponding numbers of patients were 23, 49, 57, 38, 15, and 2. These model-based estimates display the fitted linear specification, but sparse data at counts 4 and 5, the similar categorical estimates for 2 and 3–5 abnormalities, and the attenuated median quantile estimate preclude interpretation as a definitive dose–response relationship.

## 4. Discussion

In this retrospective cohort of rehabilitation outpatients after cholecystectomy, a higher routine biomarker abnormality count at the first PM&R visit was associated with a lower 12-week SPPB score after adjustment for baseline function, pain, demographic characteristics, timing, acute cholecystitis indication, diabetes, and comorbidity. The adjusted difference was approximately 0.5 SPPB points per additional abnormality, and the incremental R^2^ was 0.03, indicating modest additional explanatory value. Supportive analyses showed higher odds of low 12-week physical performance, lower odds of substantial SPPB improvement, lower grip strength, and a shorter 6-min walk distance. The pain association was of borderline statistical significance and should be interpreted cautiously. These findings add evidence that a simple cumulative profile measured at entry to post-cholecystectomy rehabilitation may capture multidomain vulnerability, but they do not establish causality or a validated prediction tool.

The construct is best understood as a multidomain routine biomarker burden rather than cumulative oxidative stress [6,7]. Nutritional reserve is reflected in part by zinc, vitamin D, and albumin; inflammatory activity by albumin and the neutrophil-to-lymphocyte ratio; and redox-related physiology by homocysteine and the antioxidant-relevant roles of zinc and vitamin D [8,9,10,23,24]. These domains overlap, and each value can be influenced by multiple clinical factors [11,22,23]. A cumulative count may therefore summarize vulnerability more pragmatically than any single result [6,7], but it should not be interpreted as a measure of nutritional deficiency alone [23,24,32]. This study did not develop a new score or assign biological weights to the components.

The association with the 12-week SPPB total score remained after adjustment for the baseline SPPB total score, although the baseline SPPB total score remained the strongest functional covariate. This finding is relevant because rehabilitation clinicians often rely on baseline performance to estimate recovery potential [17,18,25]. The present results suggest that a routinely available biomarker profile may provide modest additional information beyond baseline physical performance and clinical covariates [6,7]. The incremental R^2^ of 0.03 indicates that the additional explanatory contribution of the abnormality count was modest, which is expected for a simple count measure in a heterogeneous retrospective cohort. Thus, this finding should be interpreted as indicating a modest association rather than a strong predictive model.

The supportive outcomes provide additional context. The abnormality count was associated with higher odds of low 12-week physical performance and lower odds of substantial SPPB improvement. It was also associated with lower 12-week grip strength and a shorter 12-week 6-min walk test distance, two outcomes that reflect complementary aspects of physical function [21,26]. These analyses provide support that the primary finding was not limited to a single measure of physical performance; however, they should not be considered confirmatory because no adjustment for multiple comparisons was applied. In contrast, the association with the 12-week pain NRS score was less certain. Pain after cholecystectomy and during rehabilitation may be influenced by many factors that were not fully captured in the retrospective medical records, including gastrointestinal symptoms, activity modification, psychological distress, and analgesic use [2,3,33]. Therefore, the pain analysis should be considered exploratory and supportive rather than confirmatory.

Possible mechanisms are consistent with these overlapping domains. Reduced nutritional reserve may limit muscle performance and recovery [8,9,34]; altered homocysteine metabolism may reflect redox-related stress [10]; albumin may integrate nutrition, inflammation, and illness severity [23,32]; and an elevated neutrophil-to-lymphocyte ratio may indicate systemic inflammatory activation [11,24]. Their accumulation may identify limited physiologic reserve [5,6,18], although causal pathways cannot be inferred from this study.

Clinical implications should remain conservative. The count can be calculated from routinely available tests [22,23,24] and may support initial triage by prompting closer assessment of nutritional status, inflammation, baseline function, rehabilitation goals, and follow-up intensity [5,18,35]. It should not determine treatment, predict individual outcomes, or imply that correcting biomarker abnormalities improves physical performance. Prospective studies should evaluate additional biomarkers, recovery trajectories beyond 12 weeks, external validation in other surgical populations, and whether nutritional or rehabilitation interventions modify outcomes [5,35].

This study has several strengths. It focused on a clinically relevant outpatient rehabilitation population after cholecystectomy, used a prespecified 12-week functional endpoint, adjusted for baseline function and pain, and evaluated multiple supportive outcomes. The primary model used HC3 robust standard errors, and internal validation and robustness analyses were performed. Categorical, component-wise, leave-one-component-out, influence, interaction, and spline analyses were used to examine the stability and interpretation of the primary association.

The limitations of this study should be considered carefully. First, this retrospective, single-center observational study cannot establish causality. Second, inclusion required complete baseline biomarker data and a 12-week reassessment, and the screening log did not retain sufficient covariate data for an included-versus-excluded comparison; selection bias and limited generalizability are therefore possible. Third, surgical approach, length of stay, postoperative complications, readmission, perioperative management, and preoperative physical activity were unavailable; so, the biomarker count may partly reflect the severity or complexity of the preceding surgical course. Fourth, rehabilitation session number, frequency, components, and intensity were not quantified reliably, leaving residual confounding by treatment exposure. Fifth, assay-platform identifiers, institutional reference intervals, timestamps needed to reconstruct selection among multiple same-window results, and pre-cholecystectomy biomarker values were unavailable. The count may not reflect acute postoperative redox changes, and direct oxidative stress markers were not measured [15,16]. Sixth, dietary intake, hand dominance, analgesic timing, and assessor-level information on training, calibration, blinding, and inter-rater reliability were not available in the analytic dataset. Seventh, the unweighted continuous count assumes equal unit contributions and an approximately constant average increment; sparse counts at four and five abnormalities, similar categorical estimates, and the attenuated median quantile estimate argue against claiming a definitive dose–response relationship. Finally, supportive models were exploratory, were not adjusted for multiple comparisons, and included several covariates relative to the number of events. These limitations preclude use of the count for causal inference, individual prediction, or treatment selection.

## 5. Conclusions

In this single-center retrospective cohort, a higher routine nutritional, inflammatory, and redox-related biomarker abnormality count at the first outpatient rehabilitation visit after cholecystectomy was associated with a modestly lower 12-week SPPB score after adjustment for baseline function, pain, timing, demographic characteristics, and comorbidity. The count may provide a simple description of multidomain postoperative vulnerability for initial rehabilitation assessment and goal setting, but its incremental explanatory value was small, it is not a validated prediction or treatment selection tool, and prospective external validation is required.

## Figures and Tables

**Figure 1:**
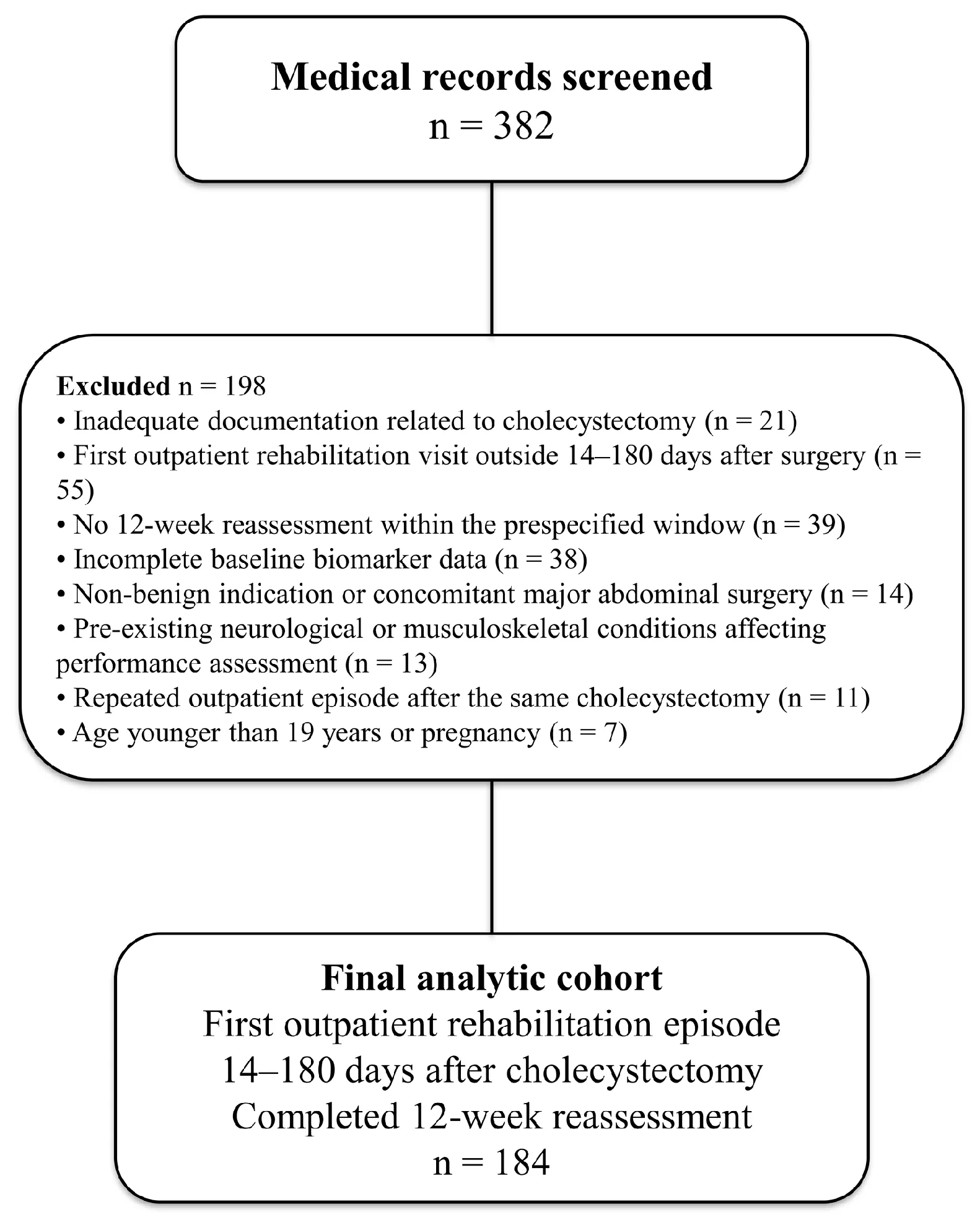
Flow of participant selection. Medical records of 382 adults who attended the physical medicine and rehabilitation (PM&R) outpatient clinic after cholecystectomy were screened. A total of 198 patients were excluded for the reasons shown, and 184 met the eligibility criteria. All included patients represented their first outpatient rehabilitation episode 14–180 days after cholecystectomy and completed the prespecified 12-week reassessment. Abbreviation: PM&R, physical medicine and rehabilitation.

**Figure 2:**
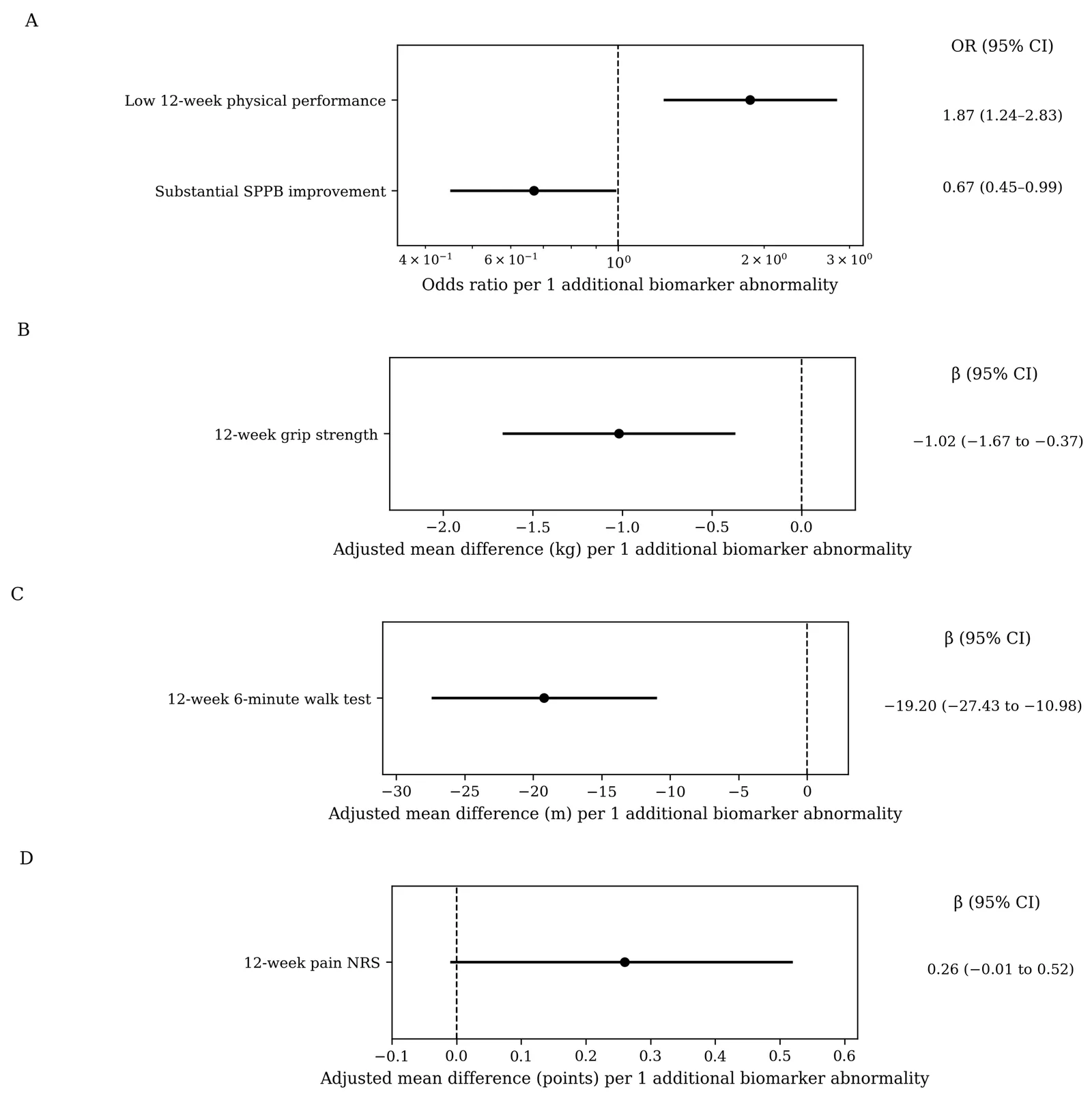
Adjusted associations between the number of routine biomarker abnormalities and 12-week outcomes. Panel (**A**) shows odds ratios for low 12-week physical performance and substantial 12-week Short Physical Performance Battery (SPPB) improvement. Panels (**B**–**D**) show adjusted mean differences for 12-week grip strength, 12-week 6-min walk test distance, and 12-week pain NRS score, respectively. Effect estimates are presented per additional routine biomarker abnormality. The logistic regression models were adjusted for baseline Short Physical Performance Battery (SPPB) total score, baseline pain NRS score, age, sex, body mass index, calendar year of the baseline visit, surgery-to-visit interval, acute cholecystitis indication, diabetes mellitus, and the Charlson Comorbidity Index. The grip strength and 6-min walk test distance models were adjusted for the corresponding baseline performance measure, baseline pain NRS score, and the same clinical covariates. The pain model was adjusted for baseline pain NRS score, baseline Short Physical Performance Battery (SPPB) total score, and the same clinical covariates. Horizontal lines indicate 95% confidence intervals. The vertical dashed reference lines represent OR = 1 in panel (**A**) and β = 0 in panels (**B**–**D**). Abbreviations: CI, confidence interval; NRS, Numeric Rating Scale; OR, odds ratio; SPPB, Short Physical Performance Battery.

**Figure 3:**
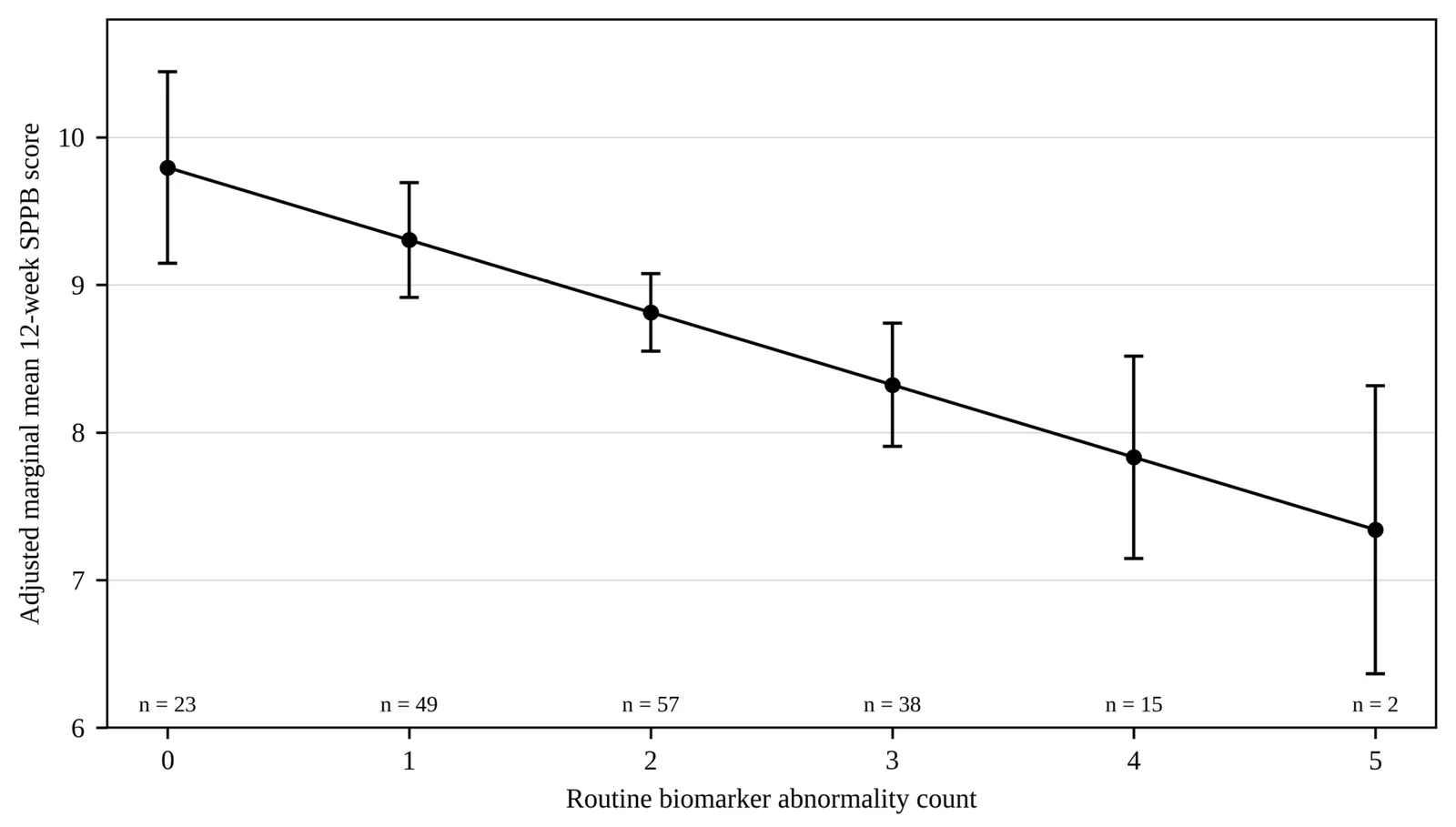
Adjusted marginal mean 12-week Short Physical Performance Battery (SPPB) total scores according to the routine biomarker abnormality count. Estimates were derived from the primary multivariable linear regression model by setting the count to each value from 0 to 5 for all participants and averaging the resulting predictions. Error bars indicate 95% confidence intervals based on HC3 robust covariance estimates. Numbers below the *x*-axis indicate the observed number of participants at each count. The plot displays the fitted linear specification and should not be interpreted as evidence of a definitive dose–response relationship. Abbreviation: SPPB, Short Physical Performance Battery.

**Table 1:** Baseline Characteristics According to the Number of Routine Biomarker Abnormalities.

Characteristic	Overall (N = 184)	0–1 Abnormalities (N = 72)	2 Abnormalities (N = 57)	3–5 Abnormalities (N = 55)	*p* Value
Age, years	63.2 ± 10.3	61.2 ± 11.3	62.8 ± 9.8	66.0 ± 9.0	0.034
Male sex	83 (45.1)	33 (45.8)	24 (42.1)	26 (47.3)	0.849
Body mass index, kg/m^2^	24.7 ± 3.2	24.8 ± 3.3	24.5 ± 3.1	24.7 ± 3.2	0.899
Calendar year of baseline PM&R visit	2021 (2019–2023)	2021 (2019–2023)	2020 (2018–2023)	2022 (2020–2023)	0.461
Surgery-to-PM&R visit interval, days	66 (44–86)	56 (36–80)	68 (39–80)	78 (60–99)	0.002
Acute cholecystitis indication	119 (64.7)	29 (40.3)	40 (70.2)	50 (90.9)	<0.001
Diabetes mellitus	43 (23.4)	7 (9.7)	14 (24.6)	22 (40.0)	<0.001
Charlson Comorbidity Index	2 (1–3)	1 (1–2)	2 (1–3)	2 (1–3)	0.499
Baseline SPPB total score	7.4 ± 1.9	8.2 ± 1.7	7.5 ± 1.7	6.2 ± 1.6	<0.001
Baseline grip strength, kg	20.5 ± 8.2	21.8 ± 9.2	21.1 ± 7.4	18.3 ± 7.3	0.048
Baseline 6-min walk test distance, m	285 ± 87	322 ± 79	288 ± 86	233 ± 72	<0.001
Baseline pain NRS score	3.1 ± 1.4	2.5 ± 1.3	3.0 ± 1.1	4.0 ± 1.4	<0.001

Values are presented as mean ± standard deviation, median (interquartile range [IQR]), or *n* (%). *p* values were calculated using one-way analysis of variance, the Kruskal–Wallis test, or the chi-square test, as appropriate. The number of routine biomarker abnormalities was calculated by summing zinc deficiency, vitamin D deficiency, hyperhomocysteinemia, low albumin status, and a high neutrophil-to-lymphocyte ratio. BMI, body mass index; IQR, interquartile range; NRS, Numeric Rating Scale; PM&R, physical medicine and rehabilitation; SPPB, Short Physical Performance Battery.

**Table 2:** Multivariable Linear Regression for 12-Week Short Physical Performance Battery Total Score.

Variable	β	95% CI	*p* Value
Number of routine biomarker abnormalities, per additional abnormality	−0.49	−0.80 to −0.18	0.002
Baseline SPPB total score, per 1 point	0.57	0.41 to 0.73	<0.001
Baseline pain NRS score, per 1 point	−0.06	−0.29 to 0.16	0.583
Age, per 10 years	0.01	−0.28 to 0.30	0.971
Male sex	0.51	−0.03 to 1.04	0.064
Body mass index, per 1 kg/m^2^	0.00	−0.08 to 0.09	0.916
Calendar year of the baseline PM&R visit, per year	0.08	−0.03 to 0.20	0.150
Surgery-to-PM&R visit interval, per 30 days	−0.20	−0.48 to 0.09	0.175
Acute cholecystitis indication	0.03	−0.57 to 0.63	0.924
Diabetes mellitus	−0.06	−0.97 to 0.84	0.893
Charlson Comorbidity Index, per 1 point	−0.05	−0.30 to 0.20	0.719
Model R^2^	0.46		
Adjusted R^2^	0.42		
Root mean square error	1.70		
Mean absolute error	1.39		
Overall model *p* value			<0.001
Maximum variance inflation factor	2.08		
Incremental R^2^ after adding the abnormality count	0.03		0.002
Bootstrap optimism-corrected R^2^	0.39		
Bootstrap optimism-corrected root mean square error	1.82		
Bootstrap calibration slope	0.94		

β coefficients represent adjusted mean differences in the 12-week SPPB total score. The model was fitted using multivariable linear regression with HC3 robust standard errors and adjusted for all variables shown. For binary variables, the coefficients compare the presence and absence of the characteristic, except for male sex, which was compared with female sex. Bootstrap internal validation used 5000 resamples. CI, confidence interval; MAE, mean absolute error; NRS, Numeric Rating Scale; PM&R, physical medicine and rehabilitation; RMSE, root mean square error; SPPB, Short Physical Performance Battery; VIF, variance inflation factor.

**Table 3:** Supportive Outcome Models According to the Number of Routine Biomarker Abnormalities.

Outcome	Outcome Distribution	Model Type	Adjusted Effect Estimate	95% CI	*p* Value	Model Performance
Low 12-week physical performance	101/184 (54.9)	Logistic regression	OR = 1.87	1.24–2.83	0.003	AUROC = 0.80; Brier score = 0.177
Substantial 12-week SPPB improvement	86/184 (46.7)	Logistic regression	OR = 0.67	0.45–0.99	0.042	AUROC = 0.73; Brier score = 0.207
12-week grip strength	22.5 ± 8.0 kg	Linear regression	β = −1.02 kg	−1.67 to −0.37	0.002	Adjusted R^2^ = 0.79; RMSE = 3.53 kg
12-week 6-min walk test distance	330 ± 92 m	Linear regression	β = −19.20 m	−27.43 to −10.98	<0.001	Adjusted R^2^ = 0.75; RMSE = 44.48 m
12-week pain NRS score	2.3 ± 1.7	Linear regression	β = 0.26	−0.01 to 0.52	0.054	Adjusted R^2^ = 0.37; RMSE = 1.28

Effect estimates are presented per additional routine biomarker abnormality. Odds ratios were obtained from multivariable logistic regression models. β coefficients represent adjusted mean differences from multivariable linear regression models with HC3 robust standard errors. The binary supportive models used the primary covariate set. The grip strength and 6-min walk test distance models included the corresponding baseline performance measure, whereas the pain model included the baseline pain NRS score and baseline SPPB total score. 6MWT, 6-min walk test; AUROC, area under the receiver operating characteristic curve; CI, confidence interval; NRS, Numeric Rating Scale; OR, odds ratio; RMSE, root mean square error; SPPB, Short Physical Performance Battery.

## Data Availability

The data are not publicly available due to privacy and ethical restrictions but may be available from the corresponding author upon reasonable request and institutional approval.

## Supplementary Materials

The following supporting information can be downloaded at: https://www.mdpi.com/article/10.3390/antiox15101306/s1, Table S1: Distribution of Routine Biomarkers and Abnormality Components; Table S2: Unadjusted 12-Week Rehabilitation Outcomes According to the Number of Routine Biomarker Abnormalities; Table S3: Full Coefficients of the Supportive Logistic Regression Models; Table S4: Full Coefficients of the Supportive Linear Regression Models; Table S5: Robustness Analyses for the Primary Association With the 12-Week Short Physical Performance Battery Total Score; Table S6: Component-Wise Continuous Biomarker Analyses; Table S7: Leave-One-Component-Out Analyses; Table S8: Variance Inflation Factors in the Primary Multivariable Model.

## Abbreviations

The following abbreviations are used in this manuscript:

6MWT	6-min walk test
AUROC	area under the receiver operating characteristic curve
BMI	body mass index
CI	confidence interval
HC3	heteroskedasticity-consistent type 3
IQR	interquartile range
MAE	mean absolute error
NRS	Numeric Rating Scale
OR	odds ratio
PM&R	physical medicine and rehabilitation
RMSE	root mean square error
SPPB	Short Physical Performance Battery
VIF	variance inflation factor
