# Supplementary material for: Routine Nutritional, Inflammatory, and Redox-Related Biomarker Abnormality Burden Is Associated with Lower 12-Week Physical Performance After Cholecystectomy in Rehabilitation Outpatients
Source: Antioxidants (Basel). 2026 Oct 9;15(10):1306. doi: 10.3390/antiox15101306 (PMC13701903; doi:10.3390/antiox15101306)
Supplement: Supplementary file 1 [file antioxidants-15-01306-s001.zip › antioxidants-4624404-supplementary.pdf]

Supplementary Tables

Table S1. Distribution of Routine Biomarkers and Abnormality Components.

Variable	Overall distribution	Abnormality definition	Count, n (%)
Zinc, μg/dL	76.6 ± 15.2	<70 μg/dL	68 (37.0)
25-hydroxyvitamin D, ng/mL	21.4 ± 8.7	<20 ng/mL	94 (51.1)
Homocysteine, μmol/L	12.9 ± 4.2	≥15 μmol/L	48 (26.1)
Albumin, g/dL	4.1 ± 0.3	<4.0 g/dL	60 (32.6)
Neutrophil-to-lymphocyte ratio	2.47 (1.77–3.92)	≥3.0	77 (41.8)
Number of routine biomarker abnormalities	1.9 ± 1.2; median 2 (IQR, 1–3)	Range, 0–5	
0 abnormalities			23 (12.5)
1 abnormality			49 (26.6)
2 abnormalities			57 (31.0)
3 abnormalities			38 (20.7)
4 abnormalities			15 (8.2)
5 abnormalities			2 (1.1)
High routine biomarker abnormality burden		≥3 abnormalities	55 (29.9)

Values are presented as mean ± standard deviation, median (interquartile range), or n (%), as appropriate. The number of routine biomarker abnormalities was calculated by summing the five listed component abnormalities. IQR, interquartile range; NLR, neutrophil-to-lymphocyte ratio.

Table S2. Unadjusted 12-Week Rehabilitation Outcomes According to the Number of Routine Biomarker Abnormalities.

Outcome	Overall (N = 184)	0–1 abnormalities (N = 72)	2 abnormalities (N = 57)	3–5 abnormalities (N = 55)	p value
12-week SPPB total score	8.9 ± 2.3	10.2 ± 1.6	8.5 ± 2.3	7.5 ± 2.3	<0.001
SPPB total score change from baseline to 12 weeks	1.5 ± 1.9	1.9 ± 1.7	1.0 ± 1.9	1.4 ± 2.0	0.016
Low 12-week physical performance	101 (54.9)	23 (31.9)	34 (59.6)	44 (80.0)	<0.001
Substantial SPPB improvement	86 (46.7)	38 (52.8)	25 (43.9)	23 (41.8)	0.411
12-week grip strength, kg	22.5 ± 8.0	24.6 ± 8.4	23.2 ± 7.6	18.9 ± 6.5	<0.001
Grip strength change from baseline, kg	2.0 ± 3.9	2.8 ± 4.0	2.2 ± 3.6	0.6 ± 3.9	0.008
12-week 6-minute walk test distance, m	330 ± 92	383 ± 77	338 ± 78	254 ± 73	<0.001
6-minute walk test distance change from baseline, m	45 ± 51	60 ± 46	50 ± 53	21 ± 47	<0.001
12-week pain NRS score	2.3 ± 1.7	1.8 ± 1.5	1.9 ± 1.6	3.3 ± 1.6	<0.001
Pain NRS score change from baseline	-0.8 ± 1.4	-0.7 ± 1.4	-1.1 ± 1.4	-0.7 ± 1.4	0.241

Values are presented as mean ± standard deviation or n (%). P values were calculated using one-way analysis of variance or the chi-square test, as appropriate. NRS, Numeric Rating Scale; SPPB, Short Physical Performance Battery.

Table S3. Full Coefficients of the Supportive Logistic Regression Models.

Outcome	Predictor	Odds ratio	95% CI	p value
Low 12-week physical performance	Number of routine biomarker abnormalities, per additional abnormality	1.87	1.24–2.83	0.003
Low 12-week physical performance	Baseline SPPB total score, per 1 point	0.61	0.48–0.77	<0.001

Outcome	Predictor	Odds ratio	95% CI	p value
Low 12-week physical performance	Baseline pain NRS score, per 1 point	0.99	0.74–1.33	0.962
Low 12-week physical performance	Age, per 10 years	0.99	0.68–1.46	0.979
Low 12-week physical performance	Male sex	0.88	0.44–1.77	0.719
Low 12-week physical performance	Body mass index, per 1 kg/m^2	0.99	0.89–1.12	0.921
Low 12-week physical performance	Calendar year of the baseline PM&R visit, per year	1.08	0.93–1.24	0.313
Low 12-week physical performance	Surgery-to-PM&R visit interval, per 30 days	0.97	0.66–1.43	0.885
Low 12-week physical performance	Acute cholecystitis indication	0.88	0.42–1.85	0.735
Low 12-week physical performance	Diabetes mellitus	0.99	0.28–3.47	0.983
Low 12-week physical performance	Charlson Comorbidity Index, per 1 point	1.09	0.80–1.48	0.579
Substantial SPPB improvement	Number of routine biomarker abnormalities, per additional abnormality	0.67	0.45–0.99	0.042
Substantial SPPB improvement	Baseline SPPB total score, per 1 point	0.57	0.47–0.71	<0.001
Substantial SPPB improvement	Baseline pain NRS score, per 1 point	0.68	0.52–0.90	0.006
Substantial SPPB improvement	Age, per 10 years	0.99	0.68–1.43	0.943
Substantial SPPB improvement	Male sex	1.86	0.95–3.64	0.069
Substantial SPPB improvement	Body mass index, per 1 kg/m^2	1.01	0.91–1.13	0.802
Substantial SPPB improvement	Calendar year of the baseline PM&R visit, per year	1.10	0.95–1.28	0.203
Substantial SPPB improvement	Surgery-to-PM&R visit interval, per 30 days	0.78	0.57–1.07	0.123
Substantial SPPB improvement	Acute cholecystitis indication	1.47	0.72–3.02	0.292
Substantial SPPB improvement	Diabetes mellitus	0.90	0.32–2.56	0.849
Substantial SPPB improvement	Charlson Comorbidity Index, per 1 point	1.01	0.74–1.38	0.928

Odds ratios were obtained from multivariable logistic regression models. Each model included the number of routine biomarker abnormalities, baseline SPPB total score, baseline pain NRS score, age, sex, body mass index, calendar year of the baseline PM&R visit, surgery-to-PM&R visit interval, acute cholecystitis indication, diabetes mellitus, and the Charlson Comorbidity Index. For binary variables, odds ratios compare the presence and absence of the characteristic, except for male sex, which was compared with female sex. CI, confidence interval; NRS, Numeric Rating Scale; PM&R, physical medicine and rehabilitation; SPPB, Short Physical Performance Battery.

Table S4. Full Coefficients of the Supportive Linear Regression Models.

Outcome	Predictor	β coefficient	95% CI	p value
12-week grip strength	Number of routine biomarker abnormalities, per additional abnormality	-1.02	-1.67 to −0.37	0.002
12-week grip strength	Baseline grip strength, per 1 kg	0.83	0.73 to 0.92	<0.001
12-week grip strength	Baseline pain NRS score, per 1 point	-0.11	-0.60 to 0.37	0.650
12-week grip strength	Age, per 10 years	-0.03	-0.71 to 0.66	0.939
12-week grip strength	Male sex	0.23	-1.14 to 1.59	0.744
12-week grip strength	Body mass index, per 1 kg/m^2	-0.10	-0.28 to 0.08	0.259

Outcome	Predictor	β coefficient	95% CI	p value
12-week grip strength	Calendar year of the baseline PM&R visit, per year	0.18	-0.07 to 0.43	0.156
12-week grip strength	Surgery-to-PM&R visit interval, per 30 days	0.04	-0.50 to 0.58	0.891
12-week grip strength	Acute cholecystitis indication	0.00	-1.28 to 1.29	0.996
12-week grip strength	Diabetes mellitus	0.33	-1.51 to 2.16	0.728
12-week grip strength	Charlson Comorbidity Index, per 1 point	0.13	-0.41 to 0.67	0.643
12-week 6-minute walk test distance	Number of routine biomarker abnormalities, per additional abnormality	-19.20	-27.43 to −10.98	<0.001
12-week 6-minute walk test distance	Baseline 6-minute walk test distance, per 1 m	0.74	0.64 to 0.84	<0.001
12-week 6-minute walk test distance	Baseline pain NRS score, per 1 point	-2.46	-8.07 to 3.16	0.391
12-week 6-minute walk test distance	Age, per 10 years	-2.67	-11.95 to 6.61	0.573
12-week 6-minute walk test distance	Male sex	-0.61	-15.07 to 13.84	0.934
12-week 6-minute walk test distance	Body mass index, per 1 kg/m^2	0.29	-1.69 to 2.27	0.776
12-week 6-minute walk test distance	Calendar year of the baseline PM&R visit, per year	1.47	-1.71 to 4.64	0.365
12-week 6-minute walk test distance	Surgery-to-PM&R visit interval, per 30 days	3.01	-3.12 to 9.14	0.336
12-week 6-minute walk test distance	Acute cholecystitis indication	-3.97	-19.42 to 11.48	0.614
12-week 6-minute walk test distance	Diabetes mellitus	-10.89	-34.27 to 12.50	0.362
12-week 6-minute walk test distance	Charlson Comorbidity Index, per 1 point	2.67	-3.65 to 9.00	0.408
12-week pain NRS score	Number of routine biomarker abnormalities, per additional abnormality	0.26	-0.01 to 0.52	0.054
12-week pain NRS score	Baseline pain NRS score, per 1 point	0.58	0.41 to 0.76	<0.001
12-week pain NRS score	Baseline SPPB total score, per 1 point	-0.01	-0.14 to 0.12	0.870
12-week pain NRS score	Age, per 10 years	0.07	-0.12 to 0.26	0.455
12-week pain NRS score	Male sex	0.34	-0.06 to 0.74	0.092
12-week pain NRS score	Body mass index, per 1 kg/m^2	0.01	-0.05 to 0.08	0.656
12-week pain NRS score	Calendar year of the baseline PM&R visit, per year	0.04	-0.05 to 0.12	0.372
12-week pain NRS score	Surgery-to-PM&R visit interval, per 30 days	0.06	-0.11 to 0.23	0.501
12-week pain NRS score	Acute cholecystitis indication	-0.43	-0.89 to 0.02	0.063
12-week pain NRS score	Diabetes mellitus	0.01	-0.63 to 0.66	0.970
12-week pain NRS score	Charlson Comorbidity Index, per 1 point	0.03	-0.15 to 0.22	0.715

β coefficients represent adjusted mean differences in the corresponding 12-week outcomes. The models were fit using multivariable linear regression with HC3 robust standard errors. The grip strength and 6-minute walk test distance models included the corresponding baseline performance measure and did not include the baseline SPPB total score, whereas the pain model included the baseline pain NRS score and the baseline SPPB total score. For binary variables, coefficients compare the presence and absence of the characteristic, except for male sex, which was compared with female sex. CI, confidence interval; NRS, Numeric Rating Scale; PM&R, physical medicine and rehabilitation; SPPB, Short Physical Performance Battery.

Table S5. Robustness Analyses for the Primary Association With the 12-Week Short Physical Performance Battery Total Score.

Analysis	Model/output	Effect estimate	95% CI	p value	Model performance/notes
Primary continuous model	Abnormality count, per additional abnormality	β = −0.49	-0.80 to −0.18	0.002	Adjusted R^2 = 0.42; RMSE = 1.70
Categorical abnormality model	2 abnormalities vs. 0–1 abnormalities	β = −1.09	-1.78 to −0.41	0.002	Reference: 0–1 abnormalities
Categorical abnormality model	3–5 abnormalities vs. 0–1 abnormalities	β = −1.08	-1.94 to −0.21	0.015	Reference: 0–1 abnormalities

Analysis	Model/output	Effect estimate	95% CI	p value	Model performance/notes
Trend across abnormality count	Abnormality count, per additional abnormality	β = −0.49	-0.80 to −0.18	0.002	Same covariate set as the primary model
Median quantile regression	Abnormality count, per additional abnormality	β = −0.19	-0.58 to 0.20	0.339	Median regression
Ordinal logistic regression	Higher 12-week SPPB tertile	OR = 0.63	0.43–0.91	0.013	Tertiles: 3–7, 8–10, and 11–12
Conventional standard errors	Abnormality count, per additional abnormality	β = −0.49	-0.80 to −0.18	0.002	Ordinary least-squares standard errors
HC3 robust standard errors	Abnormality count, per additional abnormality	β = −0.49	-0.80 to −0.18	0.002	Primary inference
Bootstrap internal validation	Abnormality count, bootstrap distribution	β = −0.48	-0.79 to −0.19		Optimism-corrected R^2 = 0.39; RMSE = 1.82; calibration slope = 0.94
Influence diagnostics	After excluding observations with Cook's distance >4/N	β = −0.40	-0.69 to −0.12	0.005	N = 177; observations with Cook's distance >4/N: 7; maximum Cook's distance = 0.060
Centered interaction model	Abnormality count × surgery-to-PM&R visit interval	β = 0.12	-0.07 to 0.31	0.233	Interaction term
Centered interaction model	Main effect of abnormality count after centering	β = −0.48	-0.79 to −0.18	0.002	Maximum VIF = 2.08
Calendar-year-excluded model	Abnormality count, per additional abnormality	β = −0.48	-0.78 to −0.17	0.002	Change in β relative to the primary model: −0.01
Spline-adjusted model	Abnormality count with spline terms for age and surgery-to-PM&R visit interval	β = −0.46	-0.77 to −0.14	0.005	Adjusted R^2 = 0.42
Acute cholecystitis-excluded analysis	Abnormality count, per additional abnormality	β = −0.73	-1.16 to −0.31	<0.001	N = 65; adjusted R^2 = 0.43

The primary outcome was the 12-week SPPB total score. Unless otherwise specified, the models were adjusted for the baseline SPPB total score, baseline pain NRS score, age, sex, body mass index, calendar year of the baseline PM&R visit, surgery-to-PM&R visit interval, acute cholecystitis indication, diabetes mellitus, and the Charlson Comorbidity Index. Bootstrap internal validation used 5,000 resamples. The acute cholecystitis-excluded analysis was exploratory because of the reduced sample size. CI, confidence interval; PM&R, physical medicine and rehabilitation; RMSE, root mean square error; SPPB, Short Physical Performance Battery; VIF, variance inflation factor.

Table S6. Component-Wise Continuous Biomarker Analyses.

Biomarker	Scaling	β coefficient for the 12-week SPPB total score	95% CI	p value	Adjusted R^2
Zinc	Per 10 μg/dL	0.05	-0.14 to 0.25	0.587	0.39
25-hydroxyvitamin D	Per 10 ng/mL	0.22	-0.10 to 0.55	0.171	0.40
Homocysteine	Per 5 μmol/L	-0.35	-0.74 to 0.03	0.070	0.40
Albumin	Per 0.5 g/dL	0.23	-0.26 to 0.72	0.352	0.39
Log-transformed neutrophil-to-lymphocyte ratio	Per 1-unit increase	-0.15	-0.78 to 0.49	0.652	0.39

Each biomarker was entered into a separate model in place of the abnormality count and adjusted for the primary covariate set. β coefficients represent adjusted mean differences in the 12-week SPPB total score for the specified biomarker scaling. CI, confidence interval; NLR, neutrophil-to-lymphocyte ratio; SPPB, Short Physical Performance Battery.

Table S7. Leave-One-Component-Out Analyses.

Omitted component	Exposure used	β coefficient	95% CI	p value	Adjusted R^2
Zinc deficiency	4-item abnormality count	-0.42	-0.73 to −0.10	0.010	0.41
Vitamin D deficiency	4-item abnormality count	-0.46	-0.79 to −0.13	0.006	0.41
Hyperhomocysteinemia	4-item abnormality count	-0.39	-0.73 to −0.06	0.022	0.41
Lower albumin status	4-item abnormality count	-0.36	-0.71 to 0.00	0.050	0.40

Omitted component	Exposure used	β coefficient	95% CI	p value	Adjusted R^2
High neutrophil-to-lymphocyte ratio	4-item abnormality count	-0.60	-0.94 to −0.25	<0.001	0.43

For each row, the listed component was omitted, and a 4-item abnormality count was entered into the primary multivariable model. β coefficients represent adjusted mean differences in the 12-week SPPB total score per additional remaining abnormality. CI, confidence interval; SPPB, Short Physical Performance Battery.

Table S8. Variance Inflation Factors in the Primary Multivariable Model.

Variable	Variance inflation factor
Number of routine biomarker abnormalities, per additional abnormality	2.08
Diabetes mellitus	1.95
Charlson Comorbidity Index, per 1 point	1.55
Baseline SPPB total score, per 1 point	1.43
Baseline pain NRS score, per 1 point	1.42
Age, per 10 years	1.33
Acute cholecystitis indication	1.28
Body mass index, per 1 kg/m^2	1.12
Surgery-to-PM&R visit interval, per 30 days	1.10
Calendar year of the baseline PM&R visit, per year	1.06
Male sex	1.03

Variance inflation factors were calculated for the primary multivariable linear regression model. PM&R, physical medicine and rehabilitation; SPPB, Short Physical Performance Battery.
